# A positive role for yeast extrachromosomal rDNA circles?

**DOI:** 10.1002/bies.201200037

**Published:** 2012-06-18

**Authors:** Anthony M Poole, Takehiko Kobayashi, Austen R D Ganley

**Affiliations:** 1) School of Biological Sciences, University of CanterburyChristchurch, New Zealand; 2) Division of Cytogenetics, National Institute of GeneticsMishima, Japan; 3) Institute of Natural Sciences, Massey UniversityAuckland, New Zealand

**Keywords:** mitochondria, petite, retrograde response, ribosomal DNA, yeast

*TAR1* (transcript antisense to ribosomal RNA) is a young gene, located antisense to the 25S rRNA gene in *Saccharomyces cerevisiae* 1. The ribosomal DNA (rDNA) exists as ∼150 tandem repeats 2, making *TAR1* the most abundant protein-coding gene in yeast. Oddly, *TAR1* is normally silenced by Sir2p, a repressor of RNA polymerase II (pol-II)-transcribed genes 3. Recent reports suggest Tar1p protein is localised to the inner mitochondrial membrane 4, interacts with Coq5p (a protein involved in coenzyme Q synthesis [5]), and can maintain oxidative phosphorylation capacity 5. Direct elucidation of *TAR1* function is lacking however, as available observations derive from monitoring a single, modified *TAR1* copy 5. This may not be representative of the majority of genomic copies, and some of these results are in conflict with previous reports on rDNA Pol-II transcript expression 3, 6, 7. Given difficulties in probing the function of a multi-copy antisense gene, we examine available data in an effort to better understand the role of *TAR1*.

We propose that *TAR1* ameliorates the behaviour of selfish yeast mitochondrial mutants first identified over fifty years ago. The location of *TAR1* in the ribosomal DNA (rDNA) repeat array is crucial to our model, as this means it is also present on extra-chromosomal ribosomal circles (ERCs). ERCs are rDNA repeats that have ‘popped out’ of the chromosome by intra-chromatid recombination, and exist in the cell as plasmid-like circular DNA (Fig. 1). ERC generation is thought to curtail replicative (though not chronological) lifespan 8, 9. However, as well as accumulating in old cells, they also accumulate in yeast with defective mitochondria 10.

**Figure 1:**
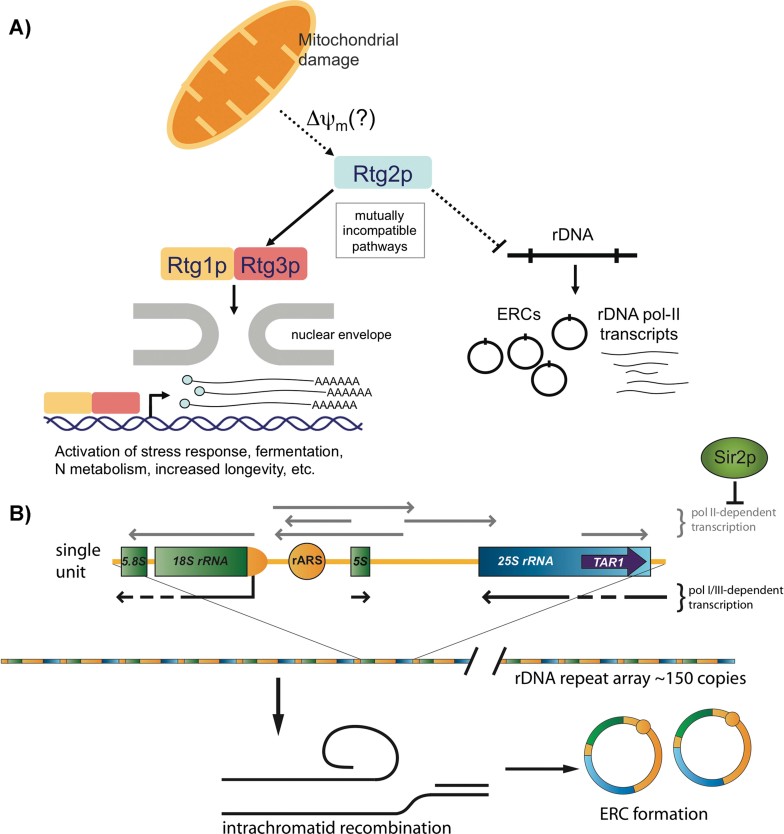
Relationship between the retrograde response and rDNA. **A:** Simplified view of the retrograde response. The retrograde response enables yeast cells to detect and respond to mitochondrial damage. In brief, some signal, possibly a drop in mitochondrial membrane potential (Δ*ψ*_m_), is transduced by the retrograde response protein, Rtg2p. Rtg2p activates a downstream transcription factor (Rtg1/3p), which leads to activation of suites of genes involved in stress response, nitrogen metabolism, fermentation pathways, and lifespan extension. At the same time, Rtg2p-dependent activation of these processes results in release of Rtg2p-dependent suppression of ERC production/rDNA pol-II silencing. Note that the figure only shows the key features of the retrograde response. Recent reviews on this topic 11, 12 give more detailed descriptions. **B:** rDNA structure and ERC formation. At the top of the panel, a single rDNA repeat unit is shown. The four ribosomal RNA (rRNA) genes found in yeast are shown as boxes and the spacer regions as orange lines, along with the *rARS* (rDNA replication origin) and the *Tar1* gene antisense to the 25S rRNA gene. The rRNA genes are transcribed by pol-I and -III, and these transcripts are depicted as black arrows below the rDNA unit. A number of pol-II transcripts have also been detected in the rDNA, and these are shown as grey arrows above the rDNA unit. These pol-II rDNA transcripts are normally heavily silenced, with Sir2p thought to play a key role. The tandem-repeat structure of the rDNA is shown beneath the single unit. Formation of ERCs is shown at the bottom of the graphic. During replication, double-strand breaks are repaired by homologous recombination, and if the end pairs with an rDNA unit on the same chromatid (intra-chromatid recombination), a unit (or unit multimer) is ‘popped’ out of the array to form an ERC. ERCs are maintained as they have a replication origin.

Significantly, ERC accumulation follows activation of the retrograde response pathway in yeast, upon mitochondrial dysfunction (Fig. 1) 11, 12. This enables survival despite diminished respiration capacity. The extent of the response corresponds to the level of mitochondrial dysfunction 13. The downstream effect of the retrograde response is upregulation of mitochondrial damage, nuclear-encoded metabolic, and stress response genes, enabling yeast to grow on fermentable carbon sources 11, 12. It also extends lifespan.

Our proposal resolves the seemingly paradoxical outcomes of the retrograde response: on one hand it extends lifespan, yet it also generates lifespan-shortening ERCs that have no known role in this response 11, 12. These lifespan effects are paradoxical only if ERCs serve solely as senescence factors 8. We propose that ERCs have a positive function: suppressing the ability of selfish mitochondrial mutants to overrun populations of sexually reproducing yeast by upregulating *TAR1* expression. Available experimental evidence supports this interpretation, which, if correct, indicates that the effect of the retrograde response on lifespan is but a side effect of ERC production, the primary aim of which is preventing the spread of respiration-deficient mitochondria.

## The retrograde response triggers changes in rDNA

Although the role of the retrograde response in alleviating mitochondrial dysfunction is well understood, it was first discovered via its effect on rDNA. Some mitochondrial mutants stimulate production of a pol-II-dependent non-coding transcript from the rDNA spacer region 6, 7, 14, but no function has been attributed to this phenomenon.

The retrograde response is also involved in ERC formation. The key retrograde response protein, Rtg2p, normally suppresses ERC formation, but upon detection of mitochondrial dysfunction, Rtg2p derepresses ERC formation 10 (Fig. 1A). In addition, pol-II-dependent transcription in the rDNA stimulates unequal recombination 15 and ERC production. Rtg2p may thus regulate ERC production by regulating pol-II-dependent rDNA transcription. We propose that these unexplained retrograde response-induced changes in the rDNA act to stimulate expression of Tar1p (Fig. 1B), suppressing genetic conflict between yeast mitochondria.

## Biparental inheritance of mitochondria creates conditions for genetic conflict

Two features of mitochondria in *Saccharomyces cerevisiae* are unusual. First, unlike many species that absolutely require oxidative respiration, yeast can lose part or all of its mitochondrial genome. Yeast unable to respire exhibit a small-colony ‘petite’ phenotype. Petites arise at a frequency of ∼1%, yet appear rare in natural populations owing to their growth disadvantage under aerobic conditions 16. Second, unlike most eucaryotes, yeast mitochondria can be inherited biparentally 17. This creates potential for genetic conflict between non-identical parental mitochondria, whereas uniparental inheritance (as in mammals) eliminates the opportunity for this conflict 18. It is assumed that biparental inheritance is tolerated in yeast because high levels of inbreeding 19 reduce opportunities for conflict to arise 18. However, recent studies have documented significant rates of outcrossing in human-associated populations of yeast 20–22. Importantly, mitochondrial genetic conflict is well known in yeast: ‘hypersuppressive’ mitochondrial petites show a transmission advantage when crossed with cells harbouring wild-type mitochondria such that the progeny will preferentially inherit the hypersuppressive mitochondria 23–25. Transmission of hypersuppressive mitochondria can ‘drive’ to 100% in such crosses 23, 26. This transmission bias creates potential for conflict between the mitochondrial and nuclear genomes, as hypersuppressive mitochondrial genomes are favoured in the short term while the nuclear genome is disadvantaged. If hypersuppressive mitochondrial DNA (mtDNA) spreads rapidly, selection would favour the appearance of nuclear-encoded modifiers that reduce or eliminate drive of hypersuppressive mitochondria. We propose that *TAR1* acts as such a modifier.

## The retrograde response increases *TAR1* expression during mitochondrial genetic conflict

Our proposal derives from findings that *TAR1* is under the control of the retrograde response and that Tar1p is targeted to mitochondria 1, 4, 5. Like other rDNA pol-II transcripts 6, 7, *TAR1* expression is pol-II-dependent and normally silenced via Sir2p 1. Therefore, silencing of *TAR1* should be lifted by activation of the retrograde response. Additionally, the location of *TAR1* antisense to the rDNA indicates that, when ERCs are produced via the retrograde response 10, the copy number of *TAR1* will also increase. Crucially, petite mitochondria (including hypersuppressives) activate the retrograde response. Therefore, increases in *TAR1* expression and copy number occur at precisely the times when a suppressor of mitochondrial conflict would be expected to act.

The retrograde response may thus have two regulatory roles: coordinating gene expression following mitochondrial damage, and suppression of the transmission advantage enjoyed by hypersuppressive mitochondria. This annuls the paradox of why the retrograde response produces both life-extending and life-shortening effects: these are separate genetic outcomes of different arms of the retrograde response. We now consider how these observations fit a model wherein *TAR1* suppresses the transmission advantage of hypersuppressive mitochondria.

## A model for Tar1p suppression of drive

‘Drive’ in the yeast mitochondrial system means the ability of one mitochondrial type to be preferentially transmitted or to subsequently overrun daughter cells if two mitochondrial types are present. If *TAR1* modifies drive, what is its mode of action? The propensity for hypersuppressives to drive may stem from a mtDNA replicative advantage. Hypersuppressive mtDNA carries many origins of replication (*ori*), which may lead to monopoly of the replication apparatus when hypersuppressives are crossed with strains harbouring wild-type mitochondria 23, 27. Indeed, hypersuppressiveness depends on the presence of a functional RNA polymerase promoter sequence contained within active *oris* 26 that is needed for mtDNA replication.

Hypersuppressive mtDNA genomes are shorter than wild-type and carry higher numbers of *ori* sequences. Consequently, any nuclear-encoded modifier ought to operate in a dose-dependent manner to counteract mtDNA overreplication. *TAR1* is a strong candidate for such a modifier for three reasons. First, rDNA copy number varies within yeast populations 28, both on chromosomes and through ERC copy number variation. Second, rDNA copy number is modulated: hypersuppressive petites elicit the retrograde response, leading to ERC production 10 and hence *TAR1* copy number increase. Third, pol-II dependent *TAR1* transcription is normally silenced by Sir2p; this silencing is reduced in petites 6, 7. These observations suggest a two-tiered mechanism for *TAR1* upregulation via the retrograde response: *TAR1* copy number increases through ERC formation, and pol-II-dependent transcription increases, perhaps specifically on ERCs 14, increasing Tar1p production.

Our model predicts a dynamic competition between nuclear *TAR1* copy number/expression and *ori* sequence copy number in hypersuppressive mitochondria. Whether hypersuppressive petite mtDNA transmission is suppressed depends upon the relative dosage of Tar1p and *ori* sequences. Similar phenomena have been seen in other cases of drive 29.

**Figure 2:**
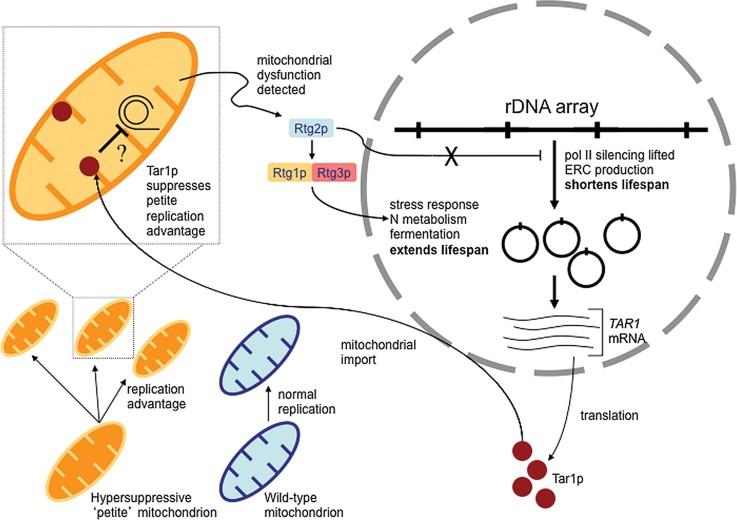
Tar1p as a putative suppressor of hypersuppressive mitochondrial transmission advantage. Upon activation of the retrograde response pathway, Rtg2p stimulates Rtg1/3p-dependent transcription. At the same time, Rtg2p-dependent suppression of ERC formation ceases and rDNA pol-II transcription is stimulated. We propose that this process upregulates expression of the rDNA/ERC-encoded *TAR1* gene. Our model is that Tar1p, which is known to be mitochondrially imported, acts to suppress the replication or transmission advantage that petite mitochondria (in particular hypersuppressive petites) have over wild-type mitochondria.

If *TAR1* does suppress drive, it presumably acts to reduce the replicative advantage of hypersuppressive mitochondria or prevent transmission to buds (Fig. 2). Interestingly, replication of hypersuppressive mtDNA occurs via single-stranded circular DNA intermediates not produced during wild-type replication 26. This difference suggests a mechanism by which Tar1p may distinguish between hypersuppressive and wild-type mtDNA.

Under our model, *TAR1* function is restricted to drive events. As not all mitochondrial dysfunction is associated with hypersuppressive petites, retrograde response-dependent ERC production can lead to *TAR1* copy number increases in the absence of hypersuppressive mitochondria. One cost associated with the retrograde response-stimulated increase in ERC production may therefore be accelerated ageing 8.

## Plausibility of stepwise evolution of drive suppression at the rDNA locus

Multicopy rDNA arrays provide a broad target for the emergence of mutants 30 and, coupled with concerted evolution in the array 2, could lead to rapid fixation of a favourable mutant (i.e. a *TAR1*-bearing rDNA repeat unit). ERC production would already have been a byproduct of rDNA array copy number maintenance, and the rDNA locus would have already been subject to Sir2p-dependent pol-II transcription silencing. We envisage that the *TAR1* open reading frame emerged by chance (other overprinted genes are known at the rDNA locus 31) and acquired suppression of drive function. Selection would then have favoured retrograde response-dependent control of ERC production/rDNA pol-II transcription. Interestingly, *TAR1* is present in *Kluyveromyces lactis* 4, which cannot form petites. However, ability to form petites is highly labile across hemiascomycetous yeasts 32, and we therefore suggest *TAR1* evolved in an ancestral petite-forming lineage.

## Experimental tests

Our model, in which we propose that *TAR1* reduces the transmission advantage of hypersuppressive mtDNA, potentially explains the connection between the retrograde response and ERC production/rDNA pol-II transcription. If true, we predict that a *TAR1* knockout will not exhibit a detrimental phenotype, other than any that may arise as a side effect of deleting an overprinted gene.

If *TAR1* is a drive suppressor, its effects should be observed postzygotically. We predict that increases in *TAR1* copy number and/or expression level would suppress the transmission advantage observed for hypersuppressives crossed with wild-type, *provided* Tar1p levels are sufficient to counteract the increased *ori* sequence copy number in hypersuppressive mitochondria. Consequently, petite hypersuppressivity should drop in crosses where *TAR1* is overexpressed. In a *tar1Δ* knockout, we predict that suppressive petite strains will become hypersuppressive. Furthermore, s*ir2Δ* mutants should resemble a *TAR1* overexpression strain, exhibiting greater resistance to drive by hypersuppressives. This should also be observed in an *rtg2Δ* knockout, which eliminates transduction of mitochondrial dysfunction but also removes suppression of ERC function 10.

Natural variations in rDNA copy number should also affect strain susceptibility to drive; whether drive occurs will be dependent on the relative copy numbers of mtDNA *ori* sequences and rDNA operons. Spontaneous emergence of hypersuppressive petites may be more frequent in younger yeast, since ERC accumulation is a facet of ageing, and ERCs are not passed to daughters 8. That said, pol-II silencing at the rDNA increases in older cells 10 and the asymmetric segregation of ERCs breaks down in very old cells 8, so petite emergence may be more frequent in older cells. It therefore remains unclear what the combined outcome of these effects on *TAR1* expression is, and whether older and younger cells differ in their resistance to hypersuppressivity.

## Concluding remarks

Our model resolves the paradoxical role of the retrograde response in lifespan. If correct, derepressing ERC formation is integral to the retrograde response as it enables Tar1p production. We propose that Tar1p eliminates the transmission advantage of hypersuppressive petite mitochondria, and reduces fixation of petite genotypes within cell lineages. According to this model, *TAR1* dampens intragenomic conflict resulting from biparental transmission of mitochondria. *TAR1* may also serve to reduce proliferation of petite mitochondria during vegetative growth, where a single mutant mtDNA spreads to fixation within a cell or cell lineage.

T. H. Huxley proclaimed the great tragedy of science to be ‘the slaying of a beautiful hypothesis by an ugly fact’ 33. However, this is also the beauty of science; if our hypothesis leads to experimental tests and new knowledge, it will have served its purpose, whether slain or not.

## Abbreviations

ERC: extra-chromosomal ribosomal circles
mtDNA: mitochondrial DNA
*ori*: origin of replication
pol-II: RNA polymerase II
rDNA: ribosomal DNA
TAR1: transcript antisense to ribosomal RNA 1
